# Successfully transfected primary peripherally mobilized human CD34+ hematopoietic stem and progenitor cells (HSPCs) demonstrate increased susceptibility to retroviral infection

**DOI:** 10.1186/s12985-020-1297-3

**Published:** 2020-02-10

**Authors:** Jeffrey Sebrow, Stephen P. Goff, Daniel O. Griffin

**Affiliations:** 1 Department of Biochemistry and Molecular Biophysics, Columbia University Medical Center, 701 West 168th Street, HHSC 1310, New York, NY 10032 USA; 2 HHMI, Department of Biochemistry and Molecular Biophysics, and Department of Microbiology and Immunology, Columbia University Medical Center, New York, NY 10032 USA; 3 Department of Medicine, Division of Infectious Diseases, Columbia University, College of Physicians and Surgeons, New York, NY 10032 USA

**Keywords:** Transfection, Transduction, CD34 +, Stem cells, Progenitor cells

## Abstract

Transfection, the process of introducing purified nucleic acids into cells, and viral transduction, viral-mediated nucleic acid transfer, are two commonly utilized techniques for gene delivery in the research setting. Transfection allows purified nucleic acid to be introduced into target cells through chemical-based techniques, nonchemical methods or particle-based methods, while viral transduction employs genomes or vectors based on adenoviruses, retroviruses (e.g. lentiviruses), adeno-associated viruses, or hybrid viruses. Transfected DNAs are often tested for potential effects on subsequent transduction, but it is not clear whether transfection itself rather than the particular nucleic acid being introduced might impact subsequent viral transfection. We observed a significant association between successfully transfected mobilized peripheral blood CD34+ human stem and progenitor cells (HSPCs) and permissiveness to subsequent lentiviral transduction, which was not evident in other cells such as 293 T cells and Jurkat cells. This association, apparently specific to CD34+ human stem and progenitor cells (HSPCs), is critical to both research and clinical applications as these cells are a frequent target of transfection and viral transduction owing to the durable nature of these cells in living systems. This finding may also present a significant opportunity to enhance the success of viral transduction for clinical applications.

## Main text

Transfection, the introduction of DNA into a cell by a variety of means, is widely used by researchers to deliberately genetically modify target cells. A large number of chemical and nonchemical techniques have been developed to allow for both transient and permanent transfection of target cells, including the use of calcium phosphate coprecipitates, basic polymers such as DEAE Dextran, lipophilic molecules such as lipofectamine, and electroporation [1–3]. Despite significant advances and a growing number of techniques, no method is equally effective for all cell types or even for all cells of a given cell type. Certain cell types are particularly difficult to transfect either owing to their low rate of cell division (neurons), their low level of metabolic activity (resting CD4+ T-cells), or a combination of defined and undefined cellular features (CD34+ human stem and progenitor cells (HSPCs)). One approach to improving the efficiency of introducing foreign genetic material into a target cell has been viral transduction, the use of a virus or virally-derived vector construct to mediate the delivery of the foreign genetic material. This approach has encountered the same difficulties in that no particular virus or construct is equally effective for all cell types or even for all cells of a certain cell type.

CD34+ HSPCs are a cell type of particular interest for transfection and viral transduction as these cells are a durable and lasting source of circulating hematopoietic cells in vivo. A number of different approaches have been investigated to understand the basis of the observed limitations to transduction of these cells and improve efficiency. Investigators have exposed CD34+ HSPCs to various chemicals such as Cyclosporin A, Cyclosporin H, Rapamycin, stem cell factor, thrombopoietin, Flt3 ligand, interleukins, proteasome inhibitors and other agents before transduction with various levels of success [4–14].

While the primary focus on transfection has been to study the impact on cells of the introduction of genetic material it would seem possible and even likely either that successfully transfected cells are different from cells that are resistant to successful transfection, or that transfected cells are modified in some way by the very process of being transfected [15–18]. We were specifically interested in the relationship between transfection and viral transduction of cells and decided to test lipofectamine-mediated transfection as well as electroporation for its possible effects on subsequent viral transduction using a lentiviral-based vector.

We obtained 293 T cells and Jurkat cells from ATCC, and peripherally mobilized CD34+ HSPCs from individual donors from AllCells (pre-isolated). 293 T cells were cultured in 20 mm X 100 mm Falcon standard tissue culture dishes in Dulbecco’s Modified Eagle Medium (DMEM) containing 10% Fetal Bovine Serum (FBS), penicillin, streptomycin, L-glutamine and 5% HEPES Buffer, and were passaged with trypsin using Gibco 0.25% Trypsin-EDTA (1X). Jurkat Cells were cultured in RPMI 1640 Medium containing 10% FBS. Human HSPCs were cultured in serum free media (X-VIVO 20), with stem cell factor (SCF), Flt3 ligand (FLT3L), and thrombopoietin (TPO) at 100 ng/ml. Lentiviral stocks for transduction were prepared by collecting culture supernatants from 293 T cells transfected with plasmid DNAs encoding a modified pNL4.3 HIV-1-based vector (AIDS Reagent Repository number 3418) (env^−^ vpr^−^ nef^−^) with ZsGreen replacing luciferase, and the vesicular stomatitis virus glycoprotein (VSV-G) envelope (addgene cat#12259) [19, 20]. The culture supernatants were collected, filtered through a 0.45 μm filter, DNase treated and then concentrated by ultracentrifugation at 100,000 x g at 4^0^ C for 2 h through a 25% sucrose cushion. Relative viral titer was determined through infection of permissive 293 T cell line with serial dilutions of the virus preparation, scoring for percent GFP positive by flow cytometry. For transfection we used pmCherry-C1 (Clontech cat# 632524) plasmid DNA in which a CMV promoter drives expression of the reporter. We selected to use a CMV promotor as this is a standard and effective promotor used in CD34+ cells for many applications including gene therapy targeting of human hematopoietic stem and progenitor cells (HSPCs) [21].

To test 293 T cells for effects of transfection, cells were plated in a 48-well plate at 50,000 cells per well. Twenty-four hours later the cells were transfected using lipofectamine. 100 ng of pmCherry-C1 plasmid DNA in 16 ul of Opti-MEM 1 Reduced Serum Medium (Gibco), was mixed with 2 ul of Lipofectamine 2000 at room temperature. Twenty minutes later the mixture was added to the cells in wells containing 250 ul of culture media to initiate the transfection [22]. Jurkat cells were transfected by electroporation in cuvettes. 200,000 cells were suspended in 20 ul of transfection reagent and 2 μg of pmCherry-C1 plasmid DNA was added. The cells were then electroporated using the Lonza 4D-Nuncleofector X Unit with the SE Cell Line 4D-Nucleofector Kit1 according to the Amaxa 4D-Nucleofector Protocol for Jurkat Clone E6.1 (https://bioscience.lonza.com). After transfection, 80 ul of culture media was added to the cuvette, and 50 ul of the cell suspension was removed and then placed in culture wells containing 200 ul of media. CD34+ cells were also transfected in cuvettes. 50,000 cells were suspended in 20 ul of transfection reagent, and 2 μg of pmCherry-C1 DNA were added. The cells were subjected to electroporation using the Lonza 4D-Nucleofector X Unit with the Lonza P3 Primary Cell 4D-Nucleofector X Kit S according to the Lonza Amaxa 4D-Nucleofector Protocol for unstimulated CD34+ cells (https://bioscience.lonza.com)*.* After the transfection process, 180 ul of culture media was added to each cuvette well and mixed with the transfection solution. A total of 90 ul of media/solution was removed and placed in culture wells containing 160 ul of media.

In all cases, at twenty-four hours following transfection, cells were exposed to the lentiviral supernatants [19]. The cells were transduced at various multiplicities of infection (MOI) as determined through titration on the highly permissive 293 T cell line. Infection of Jurkat and CD34+ cells was performed with spinoculation, whereby the cells were centrifuged at 800 x g for 60 min at 37C° after treatment with concentrated viral media. The 293 T cells were infected without spinoculation.

**Fig. 1:**
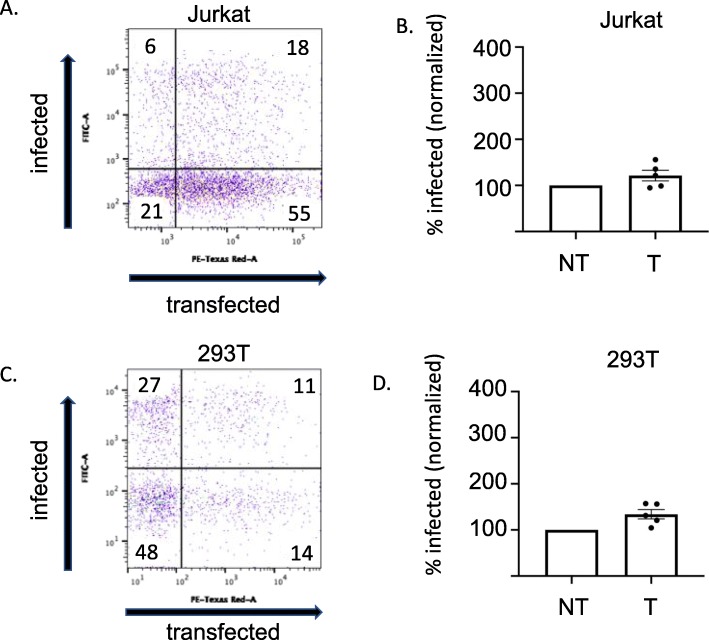
Transfection of Jurkat and 293 T cells does not show significant impacts on susceptibility to viral transduction. **a** A representative flow cytometry plot of Jurkat cells transfected with pmCherry-C1 plasmid DNA and subsequently virally transduced with ZsGreen pnl4.3 HIV-1-based vector is shown. **b** The results of several independent experiments with viral transduction performed with MOIs of 0.5 and 1 is shown (*n* = 5). Data are displayed as mean plus or minus the standard error of the mean (SEM). **c** A representative flow cytometry plot of 293 T cells transfected with pmCherry-C1 plasmid DNA and subsequently virally transduced with ZsGreen pnl4.3 HIV-1-based vector is shown. **d** The results of several independent experiments with viral transduction performed with MOIs of 0.5 and 1 is shown (n = 5). Data are displayed as mean plus or minus the standard error of the mean (SEM). Transfected (T), NonTransfected (NT)

Reporter gene expression levels were quantified using flow cytometry on a BD Biosciences LSR-II at twenty-four hours post infection for 293 T and Jurkat cells, and at 72 h post infection for CD34+ HSPCs. We gated on viable cells (based ion FSC/SSC parameters) and then read out GFP and cherry red signals. Importantly, both reporter readouts demand that the introduced DNAs enter live cells and initiate reporter gene expression. Thus, all GFP and cherry red positive signals come from viable cells and will not score any nonproductive uptake of DNA or virus. We compared the efficiency of transduction (scored as percent GFP positive) of the cells that had not been successfully transfected (cherry red low) with those that were transfected (cherry red high) using flow cytometry. We found that transfection of 293 T cells with plasmid DNA using Lipofectamine 2000 followed by viral transduction at MOIs of 0.5 and 1 did not show significant impacts on susceptibility to viral transduction (Fig. 1). We then tested Jurkat cells transfected by electroporation and again did not detect significant impacts on susceptibility to viral transduction at MOIs of 0.5 and 1 (Fig. 1).

**Fig. 2:**
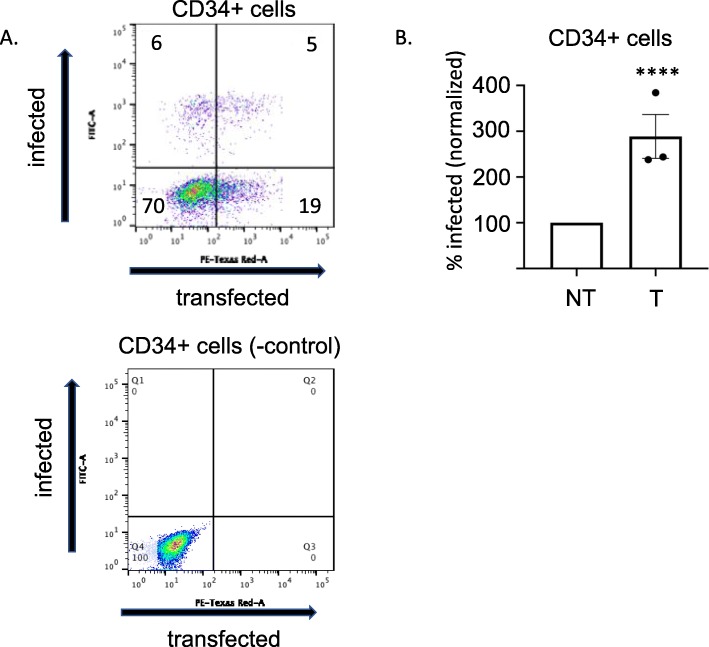
Successfully transfected CD34+ cells are significantly more permissive to viral transduction. **a** A representative flow cytometry plot of CD34+ HSPCs transfected with pmCherry-C1 plasmid DNA and subsequently virally transduced with ZsGreen pnl4.3 HIV-1-based vector is shown. **b** The results of several independent experiments with viral transduction performed at MOI 1 is shown (*n* = 3). Data are displayed as mean plus or minus the standard error of the mean (SEM). **** *P* value < 0.0001 Transfected (T), NonTransfected (NT)

In contrast, we observed that after transfection of CD34+ cells with plasmid DNA by electroporation, the successfully transfected (cherry red high) cells showed an almost 3-fold increase in the percentage of successfully transduced (GFP+) cells at an MOI of 1 (Fig. 2). Importantly, the experiments were performed multiple times with cells from multiple different patients. Data were compared for statistically relevant differences by using Student’s *t* test with two-tailed analysis (*P* value < 0.001). Similar results were obtained over the multiple replicate experiments. Importantly, the selective difference in transduction efficiency is seen even though all the cells in the preparation were subjected to the identical treatment – the only distinction is the post-experiment filtering of the cells into the successfully transfected and the unsuccessfully transfected subsets.

The results indicate a strong association between successful transfection of mobilized peripheral blood CD34+ human HSPCs and permissiveness to subsequent lentiviral transduction. It is notable that the enhanced susceptibility to transduction was only observed in HSPCs and not in transformed immortal fibroblastic or T cell lines. It is also important to note that the phenomenon cannot be attributed simply to the transfection process, because the increased susceptibility is only seen in the cell population that was successfully transfected and expressed the reporter gene, and not in the cells in the pool that were exposed to the same treatment but were not successfully transfected. The basis for the enhanced transduction is unknown, but there are several possible explanations. The cells most susceptible to transfection may represent a population subset with distinct abilities to traffic incoming nucleic acids or virion cores within the cytoplasm, into the nucleus, or to suitable locations within the nucleus for proviral integration. These steps are known to be inefficient in HSPCs [4, 5]. Close examination of the course of viral intermediates might allow a determination of any specific step in the life cycle that is enhanced in the transfected cells. Alternatively, the successful transfection event could selectively alter the cells to promote transduction. For example, the transfected cells might exhibit heightened DNA damage responses, and such activation could well alter the response to viral infections. We are working with a heterogenous cell population and we did not analyze the surface markers before and after exposure to determine if any phenotypic changes were induced or whether there was selective transduction of any specific subsets within the mixed population composed of true human HSCs (Lin − 34 + 38–90 + 45RA-dim) as well as several HPC types. It would be interesting to explore this in future experiments, as it may be that successful transfection may have differential impacts on various subsets or trigger changes in surface marker expression. Finally, those cells that express the transfected reporter driven by the CMV promoter may be a subset that are inherently especially susceptible to subsequent transduction. The characteristics of the cells actively transcribing the reporter that would account for the high sensitivity to virus are not known. In future work it would be interesting to test whether selection for expression driven by other promoters, such as the EF1a promoter, gives similar results.

Our prior work has demonstrated that the most significant block in human CD34+ cells is prior to nuclear entry and similar techniques involving quantitative PCR could be used to better understand the mechanism of this enhancement and the specific impact on various stages in the viral cycle [4, 5]. The observation of increased virus susceptibility is critical to both research and clinical applications, because CD34+ cells are widely-utilized targets in both settings. This finding is especially relevant for surveys of genes introduced into HSPCs by transfection for their potential impact on viral permissiveness. We here used an HIV-1 based lentivirus vector and it would be of interest to explore whether our findings translate into impacts on other lentiviral systems, as well as other viral delivery systems such as AAV [23]. This finding may also present a significant opportunity to enhance the success of viral transduction for clinical applications.

## Data Availability

Not applicable.

## Abbreviations

Flt3 ligand: Fms-related tyrosine kinase 3 ligand
HSCs: Human stem cells
HSPCs: Human stem and progenitor cells
MOI: Multiplicity of infection
SCF: Stem cell factor
TPO: Thrombopoietin
VSV-G: Vesicular stomatitis virus glycoprotein
